# Incisional hernias post cytoreductive surgery/peritonectomy and hyperthermic intraperitoneal chemotherapy: a systematic review and meta-analysis

**DOI:** 10.1007/s10029-023-02859-z

**Published:** 2023-08-31

**Authors:** B. M. Mac Curtain, W. Qian, H. C. Temperley, A. J. Simpkin, Z. Q. Ng

**Affiliations:** 1 School of Medicine, University of Galway, Galway, Ireland; 2 Department of Surgery, St John of God Subiaco Hospital, Subiaco, WA Australia; 3 School of Mathematical and Statistical Sciences, University of Galway, Galway, Ireland; 4 Department of General Surgery, Royal Perth Hospital, Perth, WA Australia

**Keywords:** Cytoreductive surgery, Hyperthermic/heated intraperitoneal chemotherapy, Incisional hernia, Surgical oncology

## Abstract

**Purpose:**

Cytoreductive surgery (CRS) is often combined with hyperthermic intraperitoneal chemotherapy (HIPEC) for the treatment of peritoneal tumour deposits. Considering CRS, the evidence relating the large incisions, local chemotherapy and abdominal wall trauma to incisional hernias (IH) has not been synthesized. This systematic review and meta-analysis was conducted to examine the proportion of IH present in patients post CRS and the effect HIPEC had on these rates.

**Methods:**

PubMed, EMBASE, and Cochrane Central Registry of Trials were searched up to June 2023 to examine studies relating IH and CRS plus or minus HIPEC. The most up to date PRISMA guidelines were followed. Pertinent clinical information was synthesized in tabular form. A meta-analysis reporting the pooled proportions of IH post CRS plus or minus HIPEC, the odds of IH in HIPEC versus non-HIPEC CRS and the difference in follow-up time between groups was conducted.

**Results:**

Nine studies comprising 1416 patients were included. The pooled proportion of IH post CRS was 12% (95% confidence interval (CI) 8–16%) in HIPEC and 7% (95% CI 4–10%) in non-HIPEC patients and 11% (95% CI 7–14%) overall. Previously reported rates of IH in midline laparotomy range from 10 to 30%. The odds of IH in the HIPEC was 1.9 times higher compared to non-HIPEC cohorts however this was not statistically significant (odds ratio (OR) 1.9, 95% 0.7–5.2; *p* = 0.21). There was no significant difference in average follow-up times between HIPEC and non-HIPEC cohorts.

**Conclusions:**

IH post CRS plus or minus HIPEC were in the expected range for midline laparotomies. IH in patients receiving HIPEC may occur at a greater proportion than in non-HIPEC patients, however, there were too few studies in our meta-analysis to determine this with statistical significance.

## Introduction

Cytoreductive surgery (CRS) combined with hyperthermic/heated intraperitoneal chemotherapy (HIPEC) is an effective management strategy for advanced peritoneal malignancies [1–3]. CRS aims for complete tumour removal, involving extensive peritoneal and visceral resection [4]. Once optimal cytoreduction has been achieved, HIPEC is employed intraoperatively and, in select cases, is followed by early postoperative intraperitoneal chemotherapy (EPIC) [5, 6]. However, it is associated with complications including bowel perforation, anastomotic leak and incisional hernias (IH), alongside a postoperative morbidity and mortality reported in the range 22–41% and 2–5%, respectively [7–12]. The overall incidence of IH in those undergoing laparotomy has been documented in the literature to exceed 20% [13–16]. Late morbidity and in particular the occurrence of an IH have not been well studied in those with peritoneal malignancies managed with CRS/HIPEC [17, 18].

Although the true incidence is unclear, several studies have reported an IH incidence between 7 and 17% [19–21]. CRS/HIPEC represents a complex surgical intervention of considerable duration [22]. Notwithstanding, this procedure poses a potential risk for hernia development, given several inherent factors. Primarily, a significant proportion of CRS/HIPEC patients have a history of previous abdominal surgeries, a factor well-documented to increase hernia susceptibility due to abdominal wall weakening [23, 24]. Moreover, the lengthy duration of the CRS/HIPEC procedure necessitates sizable incisions, thereby subjecting the abdominal wall to heightened stress and augmenting the likelihood of herniation [22, 25, 26]. The intraperitoneal delivery of chemotherapy during CRS/HIPEC can result in immunosuppression, further compromising abdominal wall integrity [21, 27]. Nonetheless, the precise proportion of patients developing an IH following CRS/HIPEC remains largely unexplored, as existing studies predominantly focus on short-term morbidity and long-term oncological outcomes [28].

Understanding the proportion of IH, risk factors, and outcomes related to IH post-CRS/HIPEC is essential for risk assessment, prevention, and optimal management. Further research is needed to refine preventitive strategies, standardize surgical techniques, and assess long-term outcomes to enhance patient care. The aims and learning points of this systematic review and meta-analysis is to assess the proportion of patients, risk factors and outcomes in patients who develop IH post CRS with or without HIPEC and how this information can be utilized to enhance clinical decision making for the betterment of patient outcomes and quality of life.

## Methods

### Registration and search strategy

Our search was conducted in line with the most recent preferred reporting items for systematic reviews and meta-analyses (PRISMA) recommendations [29]. Our study protocol was prospectively registered with PROSPERO under the following registration number: CRD42023432188. We conducted a search using PubMed, EMBASE and Cochrane Central Register of Controlled Trials using the search algorithms provided below on the 5th June 2023.

#### (Peritonectomy OR CRS OR cytoreductive surgery) AND (incision* AND hernia*)

**Fig. 1:**
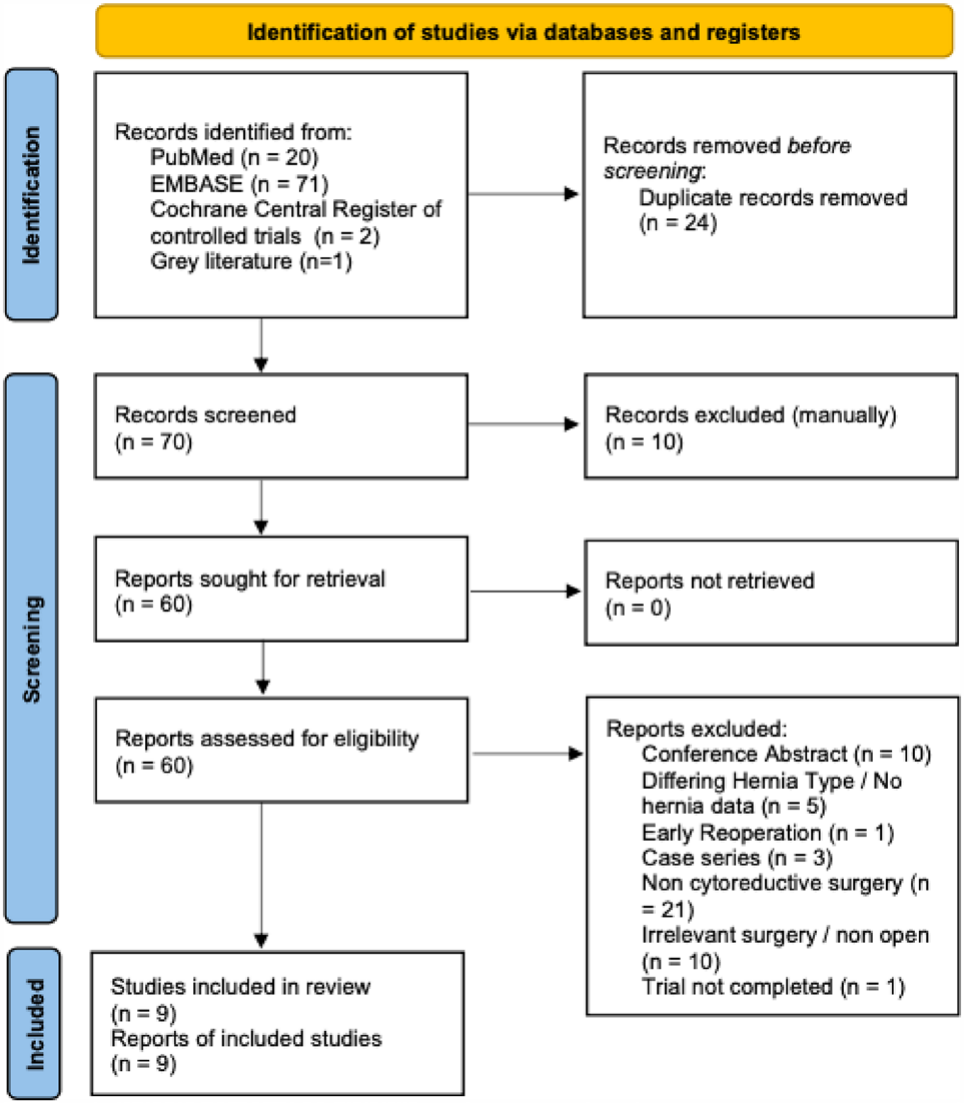
PRISMA statement for IH in CRS plus or minus HIPEC

The complete breakdown of analyzed studies can be viewed in the PRISMA diagram in Fig. 1. The bibliographies of included publications were also searched for any relevant studies.

Inclusion criteria:

- Patients aged 18 years old and above.
- Underwent CRS/Peritonectomy for oncological purposes plus or minus HIPEC.
- Prospective or Retrospective Studies.
- English language or translation available.
- Use of closure with or without a mesh support device, both primary closure and component separation techniques were acceptable.
- Reoperation cases due to tumour recurrence.
- Follow-up post CRS greater than, or equal to 12 months, on average.

Exclusion criteria:

- Laparoscopic cases.
- Case series/reports.
- Consensus statements.
- Non-IH.
- Conference abstracts.
- Non-abdominal wall related surgical procedures e.g., posterior pelvic wall CRS.
- Early reoperations as a result of initial surgery complications.
- Missing/conflicting data with no response from contacted authors.

### Identification of studies and outcomes of interest

The following population, intervention, comparison, outcome (PICO) elements were used as the basis for selecting studies [30]:

- *Population*: Patients undergoing CRS.
- *Intervention*: CRS or peritonectomy plus or minus HIPEC.
- *Comparison*: Patients whom also underwent CRS plus or minus HIPEC.
- *Outcome*: Development of IH post operation.

Studies were independently reviewed by three separate authors (BMC, WQ, HT) using Rayyan [31]. If there was any disagreement between authors, a fourth author (ZQN) was used to mediate the discussion and consensus was reached.

Our primary outcome of interest were the development of IH post CRS plus or minus HIPEC.

Secondary outcomes were risk factors and patient outcomes in relation to the development of IH post CRS plus or minus HIPEC.

### Data extraction

Relevant metrics and information were extracted using a template on Google Sheets (Mountain View, California, United States). Three independent authors (WQ, BMC, HT) were involved in the data extraction.

### Study selection

No randomized trials have been completed on the topic to the best of the author’s knowledge. Retrospective or prospective observational studies examining IH post CRS plus or minus HIPEC with at least 12 months follow-up time on average, were of interest. Where differing closure types within the same study without mesh were utilized these results were pooled into the same analysis. Only one included study reported mesh use and as such this mesh cohort was excluded from the meta-analysis [32]. One study included a small cohort (5% of patients) whom received “intraperitoneal chemotherapy” with 95% of patients not receiving any. These patients were not differentiated in terms of IH outcomes and for the purpose of this analysis all patients were classed as non-HIPEC [33]. Only first time CRS/HIPEC patients in the study authored by Wong et al. were included in our analysis due to missing data and heterogeneity of results for their repeat cohort [34].

### Risk of bias assessment

Assessment of potential biases for the non-randomised studies was assessed using a modified Newcastle–Ottawa scale risk of bias tool [35], with the results tabulated as in Table 1. This assessment tool grades each study as being ‘satisfactory’ or ‘unsatisfactory’ across various categories. We assigned stars to evaluate study quality: 7–8 stars—“very good”, 5–6 stars “good”, 3–4 stars “satisfactory” and 0–2 stars “unsatisfactory”. The critical appraisal was completed by two reviewers independently (BMC and HT), where once again a third reviewer (WQ) was asked to arbitrate in cases of discrepancies in opinion.

**Table 1:** Newcastle Ottawa risk of bias assessment for included non-randomised studies

Author	Selection				Comparability	Outcome			Quality
	Representativeness of the exposed cohort	Sample size (< 25 = no star)	Open cases only included	Ascertainment of the exposure	The subjects in different outcome groups are comparable	Assessment of outcome	Less than 10% missing data?	Average follow-up period (> 12 months)	
Boutros (2010)	✸	–	✸	✸	✸	✸	✸	–	6
Tzivanakis [63]	✸	✸	✸	✸	✸	✸	✸	✸	8
Struller [20]	✸	✸	✸	✸	✸	✸	✸	✸	8
Ravn [19]	✸	✸	✸	✸	✸	✸	✸	✸	8
Tuttle [21]	✸	✸	✸	✸	✸	✸	✸	✸	8
Parikh [32]	✸	✸	✸	✸	✸	✸	✸	✸	8
Lewcun [44]	✸	✸	✸	✸	✸	✸	✸	✸	8
Spencer [33]	✸	✸	✸	✸	✸	✸	✸	✸	8
Cascalcs Campos [10]	✸	✸	✸	✸	✸	✸	✸	✸	8
Wong [34]	✸	✸	✸	✸	✸	✸	✸	✸	8
Wenzelberg [27]	✸	✸	✸	✸	✸	✸	✸	✸	8

### Statistical analysis

We performed a proportional meta-analysis as part of this review [36]. Statistical analysis was run using Stata 17 (StataCorp. 2021. *Stata Statistical Software: Release 17*. College Station, TX: StataCorp LLC). The proportion of patients developing IH post CRS plus or minus HIPEC was pooled using the “metaprop” function within Stata [37]. 95% confidence intervals (CI) were employed and p ≤ 0.05 was considered statistically significant. Heterogeny was reported using I^2^ [37]. We considered there to be a notable degree of heterogeny if I^2^ was greater than 50% [38]. A random effects model was used due to evidence of significant statistical heterogeneity as well evidence of study design heterogeneity [39].

To assess publication bias, funnel plots were generated. These are not included in this article as recommended in the literature, due to less than 10 papers being included in the analysis, thus making it an inaccurate representation of publication bias [40]. Qualitative bias assessment was also conducted as proposed by Barker et al. as this is a proportional meta-analysis [36]. If missing data or conflicting data were found upon review of included papers authors were contacted for clarification.

The relationship between HIPEC and non-HIPEC IH proportions was examined using the “metafor” package in R v4.1 [41]. (R Core Team (2021). R: A language and environment for statistical computing. R Foundation for Statistical Computing, Vienna, Austria.URL https://www.R-project.org/), as previously described [42]. To assess whether follow-up time could be responsible for differences in IH, an independent student’s *t* test was used to examine the mean follow-up times in relation to non-HIPEC and HIPEC groups. Where studies reported a median and range the mean was estimated using the method put forward by Wan et al. [43]. If follow-up was reported at a set time point, for example one year, this was taken as the mean. If a study reported a minimum follow-up period, this was also taken as the mean for the purpose of follow-up analysis.

## Results

Our search yielded ninety four articles of which nine studies were selected for data extraction [10, 19–21, 32–34, 44]. Studies selected were published between 2014 and 2023, conducted in six countries. A total of 1416 patients were included in our analysis. Study characteristics and patient demographics are found in Table 2 and 3, respectively. All but one study was conducted retrospectively [19]. All but one study took place at a single institution [33]. One study did not specify its location but collected data from a prospectively maintained database [32]. Eight studies included patients who underwent both CRS and HIPEC. Spencer et al. included patients who underwent CRS only [33]. Cascales Campos et al. described two groups, CRS only and a group who underwent both CRS and HIPEC [10]. Patients who underwent HIPEC received variable regimens, but all with either platinum agents such as oxaliplatin/cisplatin, mitomycin or both. Pathologies were wide ranging with the majority described as ovarian cancer, peritoneal mesothelioma, colorectal cancer and appendiceal cancer. Three studies included recurrent disease [10, 20, 32]. Spencer et al. and Wong et al. reported on ovarian cancer and mesothelioma in isolation, respectively [33, 34].

**Table 2:** Study characteristics and patient demographics

Study title	Author	Year	Journal	Country	Study duration	Study design	Gender	Age	Patient numbers	Recurrent disease
Abdominal wall morbidity following cytoreductive surgery and hyperthermic intraperitoneal chemotherapy	Struller [20]	2017	Scandinavian Journal of Surgery	Germany	9 years	Retrospective	M 33%, F 66%	55 (median)	271	Recurrent ovarian cancer included
Risk factors and management of incisional hernia after cytoreduction and hyperthermic intraperitoneal chemotherapy (HIPEC) in patients with peritoneal surface malignancies	Cascales Campos [10]	2019	Springer	Spain	9 years	Retrospective	M 8% F 92%	60 (median)	282	Recurrent disease 35% in no IH group, 25% in IH group
Incidence and predictors of incisional hernia after cytoreductive surgery and hyperthermic intraperitoneal chemotherapy	Tuttle [21]	2019	International Journal of Hyperthermia	USA	15 years	Retrospective	M 42% F 58%	Age 18–49: 64 (41%) Age 50 + : 91 (59%)	155	n/a
Incisional hernia and its impact on health- related quality of life after cytoreductive surgery and hyperthermic intraperitoneal chemotherapy: a national prospective cohort study	Ravn [19]	2018	World Journal of Surgical Oncology	Denmark	9 years	Prospective	M 34% F 66%	60 (median)	152	n/a
Incisional hernia formation can be reduced following hyperthermic intraperitoneal chemotherapy with increased suture length to wound length ratio fascial closure	Lewcun [44]	2020	International Journal of Abdominal Wall and Hernia Surgery	USA	7 years	Retrospective	M 44.2% F 55.8%	58.6 (mean)	86	na
An analysis of the morbidity associated with abdominal wall resection and reconstruction after cytoreductive surgery and hyperthermic intraperitoneal chemotherapy (CRS/HIPEC)	Parikh [32]	2019	European Journal of Surgical Oncology	Australia	11 years	Retrospective	M 46% F54%	53.8 (mean)	197 (126 no mesh)	42 (31%)
Risk factors for early-occurring and late-occurring incisional hernias after primary laparotomy for ovarian cancer	Spencer [33]	2015	Obstetrics and Gynaecology	USA	6 years	Retrospective	M 0% F 100%	Age < 65 134 (71%), Age > 65 55 (29%)	189	n/a
Repeat cytoreductive surgery and heated intraperitoneal chemotherapy may offer survival benefit for intraperitoneal mesothelioma: a single institution experience	Wong [34]	2014	Annals of Surgical oncology	USA	8 years	Retrospective	M 66% F 33%	65 (median)	26	n/a
Abdominal closure with reinforcing suture decreases incisional hernia incidence after CRS/HIPEC	Wenzelberg [27]	2023	Journal of Abdominal Wall Surgery	Sweden	15 years	Retrospective	M 48% F 52%	57 (mean)	129	n/a

**Table 3:** Closure technique, HIPEC usage, IH incidence, IH repair, risk factors and outcomes. RR = relative risk, OR = odds ratio.

Study	Mesh used at index operation	HIPEC	Diagnosis	Follow-up duration (months)	Incisional hernia occurrence	Hernia repair	Risk factors	Patient outcomes
Struller	No	Y	Clinical + radiological (CT)	38 (median)	19 (7%)	12 (63%) due to clinical symptoms, but no incarcerations occurred	Multivariate (Abdominal wall rupture OR 11.682 *p* < 0.0001, presence of pseudomyxoma peritonei/mesothelioma OR 4.295 *p* = 0.022 were found to be significant risk factors for IH formation)	n/a
Cascales campos	No	Y 80% N 20%	Clinical + radiological (CT)	12 minimum	28 (10%)	11 patients (39% of those with IH) had surgical repair. Surgery for IH was only indicated in cases with an acute complication (incarceration or strangulation), in which symptoms associated with poor subjective quality of life were reported. In patients who required a new CRS and HIPEC treatment, the repair was performed during this second CRS	Univariate (pre-op chemotherapyo *p* = 0.047, HIPEC *p* = 0.038, Colon cancer primary *p* = 0.0 1). Multivariate (HIPEC OR 2.56 CI 1.57–4.31 *p* = 0.032, pre-op chemo OR1.59 CI 1.26–3.58 *p* = 0.041	n/a
Tuttle	No	Y	Clinical + radiological	24 minimum (range 2–13 years)	26 (17%)	n/a	Univariate (obesity, previous IH, pre-op chemotherapy, post op chemotherapy, were significant predictors of IH *p* < 0.001, *p* = 0.04, *p* = 0.03, *p* = 0.001, respectively.) Multivariate (Age 50–64 OR 0.08 CI 0.01–0.64, Female OR 0.09 CI 0.01–0.75, BMI > 30 OR 0.03 CI 0.01–0.37 were significant independent predictors of IH)	No statistically significant outcomes identified
Ravn	No	Y	Clinical + radiological (PET CT)	16.6 (median)	14 (9.2%) 8 developed IH within first year. (CI 2.9–10.4). 14 developed IH within 2 years (CI 5.3–14.5)	4/14 (28.6%) IH patients underwent hernia repair within the follow-up period	Age only significantly different factor between IH and non IH group. 67 vs 60, respectively *p* < 0.01	Statistically significant differences in QOL through SF-36 between IH and no IH patients in domains of *Role-physical* and *Role-emotional*
Lewcun	No	Y	Clinical + radiological	17.4 (mean)	25 (29%) (increased suture length ratio: 3 Standard fascial closure: 22)	n/a	Standard fascia closure vs 4:1 suture to wound length ratio incisional hernia rate: 34.9% vs 13% *p* = 0.048	n/a
Parikh	Yes: primary fascia closure 116 (59%), biological mesh repair 34 (17.3%), synthetic mesh repair 26 (13.2%) Component separation technique with/without mesh 21 (10.6%)	Y	n/a (not specified)	84.7 (mean)	16 (8.2%), 8 no mesh (6.3%)	n/a	Mesh repair did not show significant reduction incisional hernia incidence compared to non-mesh repair. 11.26% vs 6.34%, OR 1.77, CI 0.64–4.93,* p* = 0.271	Wound complications 21 (10.6%). Patients who required abdominal wall resection at higher risk (*p* = 0.0032)
Spencer	No	5% received intraperitoneal chemotherapy. Unsure if HIPEC	Clinical and/or radiological	24 (Stopped at 2 years post op)	15 (8%)	All IH patients reviewed by general surgeon but none required emergent repair	In 1st year of follow-up: Multivariate (Nutritional status RR 48 CI 14–165 *p* < 0.001, Suboptimal cytoreduction during surgery RR 4.3 CI 2.5–7.3 *p* < 0.001) was found significant. In second year of follow-up: Multivariate (Age > 65 RR3.5 CI 1.3–9.4 *p* = 0.01) found significant	n/a
Wong	No	Y	n/a (not specified)	12.2 (median) (range 1.4–53.8)	3 (11.5%)	n/a	n/a	n/a
Wenzelberg	No	Y	Radiological (CT)	12 + /–3 months	10 (7.8%)	n/a	Univariate: cardiovascular disease significant for IH formation *p* = 0.024	n/a

### Incisional hernia

Overall, 148 incisional hernias occurred within the included studies. Six studies diagnosed post operative IH through clinical and radiological assessment, whilst Wenzelberg et al. used CT imaging solely for diagnosis [27]. Wong et al. and Parikh et al. did not specify the diagnostic method [32, 34].

In the pooled proportion of CRS/HIPEC patients, IH occurred in 12% (95% CI 8–16%). Significant heterogeneity was found between studies (I^2 75.24%, *p* < 0.01).

### Risk factors

A wide range of risk factors were identified in their contribution to IH formation. Patient pathology was identified as a significant risk factor by Struller et al. with pseudomyxoma peritonei and peritoneal mesothelioma patients at higher risk of developing IH (OR 4.295 *p* = 0.022) [20]. Three studies found patient characteristics such as old age, female sex and increased BMI > 30 were significant risk factors [19, 21, 33]. Two studies examined closure techniques post CRS/HIPEC, and found an increased 4:1 suture to wound length ratio was beneficial for prevention of IH (*p* = 0.048), whilst the use of mesh was not effective [32, 44]. Wenzelberg described cardiovascular disease as a significant risk factor for IH formation (*p* = 0.024) [27]. Spencer et al. identified poor pre-op nutritional status as a risk factor for IH occurrence in the first year of follow-up (*p* < 0.001), whilst Cascales Campos et al. identified pre-op chemotherapy as a risk factor (*p* = 0.041) [10, 33]. Wong et al. did not describe risk factors for IH formation [34].

### Non IH reported patient outcomes

The studies included reported heterogenous outcomes. No study identified CRS with HIPEC as independent risk factors for IH formation on multivariate analysis. Patients with IH had significantly decreased quality of life compared to those who did not develop IH using the Short Form Survey-36 tool in the domains of *Role-physical* and *Role-emotional* [19]. Parikh et al. identified wound complications such as dehiscence and wound infection as significant comorbidities in patients requiring abdominal wall resection during CRS/HIPEC (*p* = 0.0032) [32]. No studies reported on overall survival outcomes relating to IH. Further information pertaining to chemotherapy regimen used is reported in Table 4.

**Table 4:** Pathology, neoadjuvant chemotherapy use, Completeness of cytoreduction score (CC) and Peritoneal cancer index (PCI) scoring, HIPEC regimen, I.p = intra-peritoneal, 5-FU - 5 fluorouracil

Study	Pathology	Neoadjuvant chemotherapy	PCI	Procedure	CC score	Temp (C)	Oxaliplatin	Mitomycin	Others
Struller	Colorectal, appendiceal, ovarian, pseudomyxoma peritonei, gastric cancer, mesothelioma, small bowel	n/a	16 (mean)	Cytoreductive	n/a	Only given for initial cisplatin dosage. 42 degrees	Since 2012 change to: Oxaliplatin 300 mg/ m^2^ intraperitoneally (i.p.) combined with 5-FU (400 mg/m^2^) intravenously (i.v.) for 30 min in colorectal cancer and pseudomyxoma peritonei patients	Mitomycin 35 mg/m^2^ (colorectal cancer, pseudomyxoma peritonei)	Cisplatin 50 mg/m^2^ for 90 min at 42 degrees (gastric/ovarian/mesothelioma). Since 2012 changed to Cisplatin (75 mg/m2 i.p.) combined with Doxorubicin (15 mg/m2 i.p.) was used in gastric cancer, recurrent ovarian cancer, and mesothelioma
Cascales campos	Ovarian, colon, appendix-pseudomyxoma, others	No IH: 31%, IH: 86%	Without IH: 10.26 With IH: 8 (mean)	Cytoreductive	CC 0 in 89% of those with no hernia. CC 1 in the remainder. CC 0 in 82% of those with incisional hernia. CC 1 in remainder	42	n/a	Y, 60 min	Cisplatin, paclitaxel. All 60 min
Tuttle	Appendiceal, colorectal, ovarian, peritoneal mesothelioma, gastric, other	Pre-op Y 30%, N 70%. Post-op Y 51% N 23% Unknown 26%	< 10: 60%, > 10: 40%	Cytoreductive	n/a	39–41	Y	Y	n/a
Ravn	Colorectal cancer, appendix cancer, pseudomyxoma peritonei, malignant peritoneal mesothelioma	Neoadjuvant 50%. Adjuvant—all patients, except patients with pseudomyxoma peritonei with low-grade neoplasia, were offered postoperative systemic adjuvant chemotherapy for 3–6 months	n/a	Cytoreductive	n/a	n/a	n/a	Y	n/a
Lewcun	Appendiceal, colon, ovarian, gastric	Systemic chemotherapy within 6 months post-op 68.6%	n/a	Cytoreductive	n/a	40.7 max temp	n/a	Majority of patients were treated with mitomycin‐C during HIPEC, although the HIPEC agents were subject to variation due to differences in tumour biology	Cisplatin and doxorubicin
Parikh	Colorectal, mesothelioma, low/high grade appendix, ovarian	n/a	< 20: 77.1%, ≥ 20: 22.9%	Cytoreductive	CC 0–1 97.4%,CC 2–3 2.6%	n/a	n/a	n/a	n/a
Spencer	Ovarian	90% patients received IV platinum and taxane in peri-operative period	n/a	Cytoreductive	n/a	n/a	n/a	n/a	IV platinum and taxane agents. Specific agent not mentioned
Wong	Mesothelioma	Y 14 (54%)	< 20: 67%, ≥ 20: 33%	Cytoreductive	CC 0–1 78%, CC2-3 22%	39–42	n/a	n/a	Cisplatin
Wenzelberg	Colon, appendiceal, redctal, peritoneal pseudomyxoma, small bowel cancer, fallopian tube cancer, malignant mesothelioma	Y 32 (24.8%)	11 (mean)	Cytoreductive	CC 0 95.9%, CC1 + CC2 4.1%	n/a	n/a	n/a	Not given

### Meta-analysis

#### Pooled proportions of IH

**Fig. 2:**
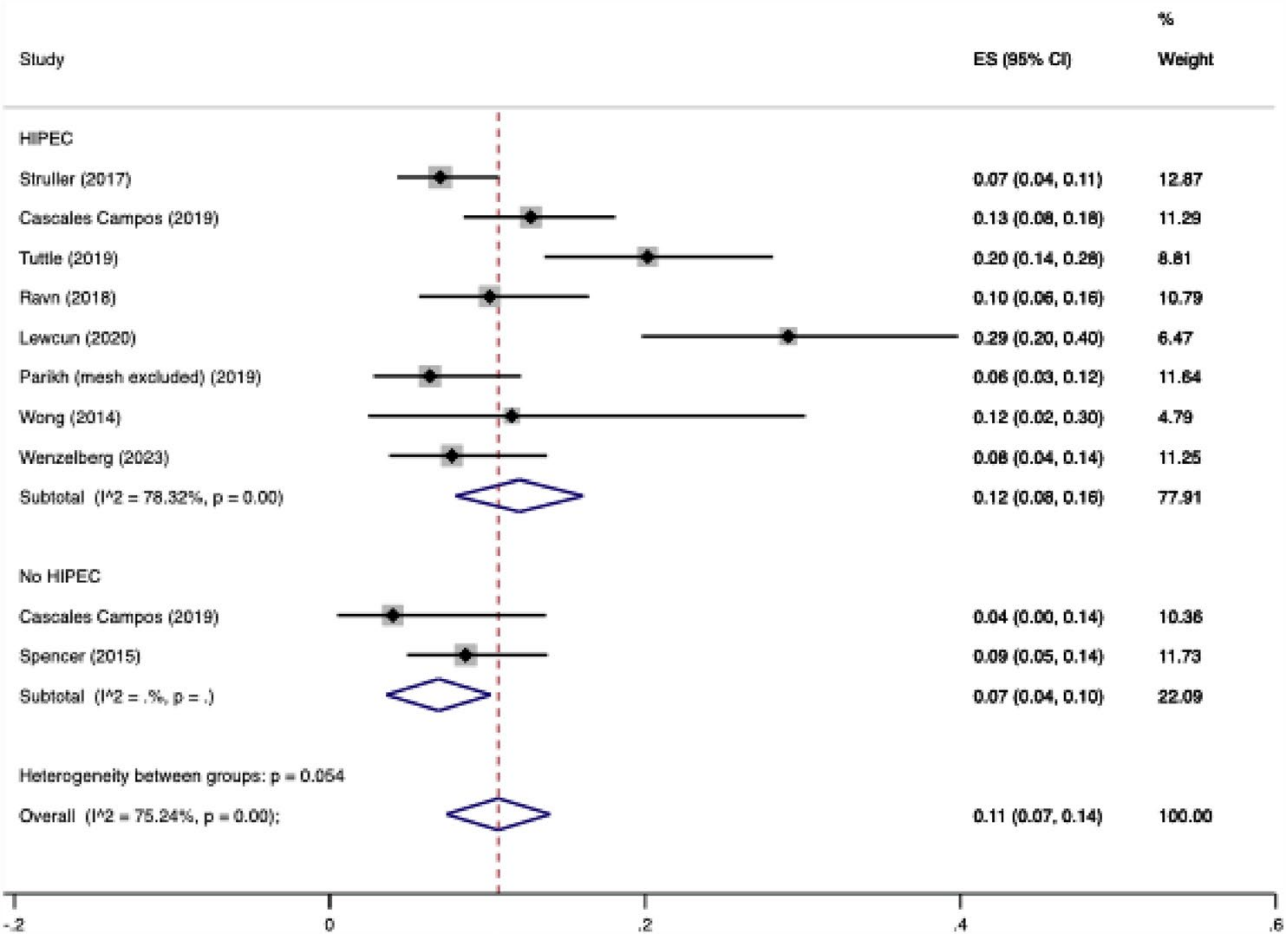
Forest plot displaying the pooled proportion of patients post CRS/HIPEC developing an IH over their respective follow-up periods

Nine studies were included in the pooled analysis. The pooled proportion of patients whom developed an IH post CRS plus or minus HIPEC. The pooled proportion of patients developing an IH in the cohort receiving HIPEC was 12% (95% confidence interval CI 8–16%). The pooled proportion of patients developing an IH in the cohort non receiving HIPEC was 7% (95% CI 4–10%). There was significant heterogeneity between studies with an I^2^ = 78.32% (*p* < 0.01). Overall, the proportion of CRS plus or minus HIPEC patients developing an IH was 11% (95% CI 7–14%). The results are visually described in Fig. 2. Of note, studies subjected patients to differing follow-up times as described in Table 3.

### Odds of IH in HIPEC and Non-HIPEC cohorts

We report an odds ratio (OR) and 95% CI relating the odds of developing an IH in patients whom underwent HIPEC compared to patients who did not. In the pooled HIPEC cohort, patients had nearly twice the odds of IH (OR = 1.9, 95% CI 0.7, 5.2) when compared to non-HIPEC cohorts. However, there is no strong evidence for this effect at a generalisable population level, since *p* = 0.21 and the CI includes 1 (a null ratio). Our interval is quite wide, with 30% lower odds of IH in HIPEC or up to 5.2 times higher odds of IH in HIPEC possible.

### Difference in follow-up times

**Fig. 3:**
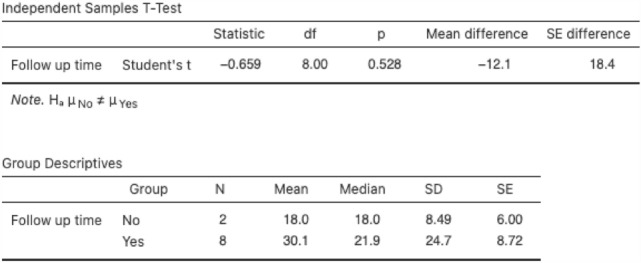
Independent samples *t* test for follow-up time (months) between HIPEC and non-HIPEC groups

An independent samples *t* test was used to examine the relationship between follow-up times in the HIPEC and non-HIPEC cohorts, as this may skew results. Results are as observed in Fig. 3.

**Fig. 4:**
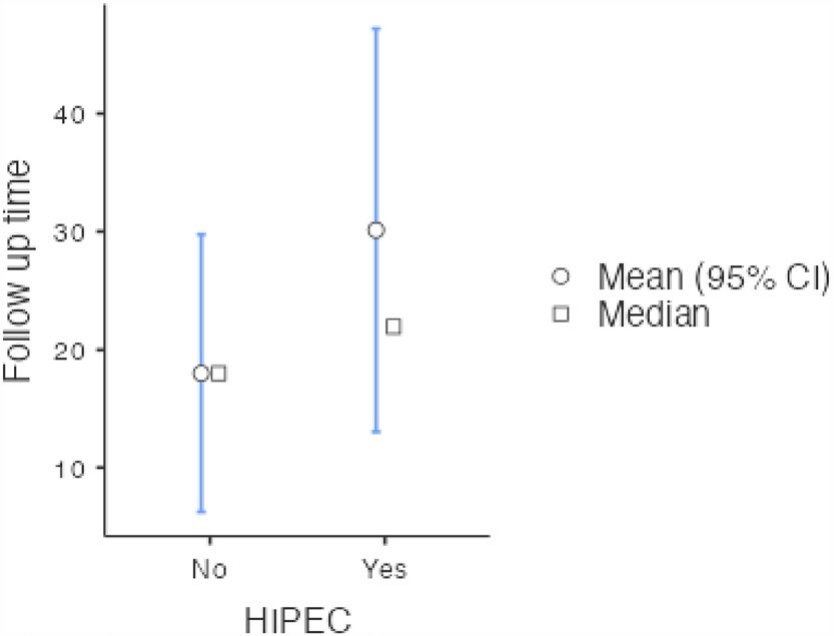
Follow-up times (months) in HIPEC versus non-HIPEC groups

We can see a mean follow-up of 18 months in the non-HIPEC group and 30.1 months in the HIPEC group. This results are not statistically significant, *p* = 0.53. This is visually illustrated in Fig. 4. Here, we can observe the CI of the two groups overlapping, and the median value, below that of the mean in the HIPEC group, possibly indicating skewed data.

## Discussion

We performed a systematic review and meta-analysis regarding the occurrence of IH post CRS plus or minus HIPEC. From our results, we report a pooled proportion of patients developing an IH of 12% in the HIPEC group, 7% in the non-HIPEC group and 11% overall. There was evidence of statistical heterogeneity in the HIPEC group and between groups. Considering the odds of developing an IH post CRS/HIPEC we reported an OR of 1.9, which was not statistically significant, indicating further research is required to determine clinical significance. These results indicate that IH may be more likely in the HIPEC group. We also observe no statistically significant difference between mean follow-up times in HIPEC or non-HIPEC groups, which can affect the rates of IH observed [45].

Rates of IH post midline laparotomy, not specifically related to CRS, of 10–30% have been described [46]. The pooled proportion of IH post CRS plus or minus HIPEC of 11% is at the lower end of expected rates. This may be due to a number of reasons, including closure technique, BMI, previous surgery, age and gender [47–50]. As well as this, the actual rates of IH post CRS plus or minus HIPEC may be higher. Considering midline laparotomy in general, only 75% of IH were seen to occur within 2 years of surgery in previous studies [48].

Beadles et al. have shown incidence rates of IH emergency repair in elderly women and men of 23.5 and 32.0 per 100,000 population in the United States, respectively [51]. This serves to highlight the impact IH can have on patient outcomes, and healthcare systems.

In an obese cohort undergoing midline laparotomy, required IH repairs rates of 29% have been reported in the literature [52], with the rate of incarcerated IH repair reported as 3.7% [53]. The expected rates of IH in midline laparotomy in conjunction with peritonectomy may be expected to be higher. Within the included studies, 11 out of 28 patients underwent surgical correction of their IH, with one surgery classed as an emergency due to incarceration [10]. Tuttle et al. reported 10 patients whom underwent surgical repair out of 26 IH [21]. 4 out of 14 IH were repaired in Ravn et al.’s publication, with one case classed as an emergency obstruction [19]. 12 out of 19 IH were repaired electively, in Struller et al.’s study [20]. 7 from 265 patients underwent non-emergency IH repair in an ovarian cancer cohort [33]. What must be considered is the benefit of CRS and HIPEC in contrast with the risks of emergency IH repair and morbidity associated with this procedure, in an immunosuppressed patient population.

Regarding ventral hernias, laparoscopic as opposed to open cases have been described as a more cost effective method of repair when hernias recur, however, all are economically costly [54]. In the case of IH repair post peritonectomy, open surgery may be the most effective option due to the fact it may no longer be possible to place a pre-peritoneal mesh. Additionally a retro-rectus approach may not be feasible if the posterior rectus sheath is resected, leaving the option of an onlay repair, which has its own complications [55, 56]. If open repair is undertaken this will further increase repair economic cost.

Our review also identified risk factors that may suggest patients are more likely to develop an IH as described in Table 3.

The primary malignancy was seen to affect IH rates post CRS/HIPEC, with pseudomyxoma peritonei and mesothelioma patients more likely to develop an IH (*p* = 0.022) [20]. A colorectal primary has also been described as a risk factor for IH by Cascales Campos et al. (*p* = 0.01), while Spencer et al. details a suboptimal CRS as a risk factor (*p* < 0.001), which may be considered a surrogate of primary cancer aggressiveness [10, 33]. Nutritional status was also reported as a risk factor for IH in one study (*p *< 0.001) [10], which is in agreement with previous literature regarding inguinal hernias [57]. Peritoneal cancer/carcinomatosis index (PCI) has been described as accurate in predicting outcomes, however, others have questioned its benefit [58, 59]. Parikh described a PCI greater than 20 as a high burden of disease, but failed to show statistical significance in relation to wound complications post CRS, however, they did not specifically analyze PCI in relation to IH [32]. Wong et al. also reported the effect of PCI on outcomes. They did not analyze PCI in relation to IH but did find PCI > 20 to correlate with overall survival [34]. Of note, our included studies did not report the effect of stoma formation on IH rates, however, previous research has shown rates of anastomotic leak and prognosis seem to be within the established range when stomas are fashioned in CRS [60, 61]. Further research relating stoma formation to IH outcomes may be clinically useful.

The use of meshes in patients with peritoneal metastases has been questioned [62]. However, the use of mesh reconstruction in patients post CRS/HIPEC/laparotomy has been shown to be safe and effective [46, 63, 64]. Only a small cohort of patients in one study included in this analysis reported mesh use [32], however, ongoing studies (ClinicalTrials.gov identifier: NCT03953365) relating to the outcomes regarding mesh use post CRS/HIPEC may further enhance patient outcomes regarding IH. One study included in our analysis did not show a IH development rate that was statistically significant between mesh and no mesh groups [32].

The major limitation of this meta-analysis is an inherent limitation of each of the included studies. The follow-up time was likely insufficient to detect all IH post surgeries. The HIPEC group had a mean follow-up of 30 months and the non-HIPEC group had follow-up of 18 months, falling short of the recommended minimum follow-up period of 36 months [45]. Another limitation is the lack of standardisation in follow-up times, and while we utilized 12 months as an inclusion minimum there is likely to be a difference in IH picked up with longer follow-up, however, in this patient cohort longer follow-up may be difficult due to patient mortality prior to IH development [19–21]. Previously described limitations of the statistical methods are also valid [39]. Due to the low volume of papers describing non-HIPEC cohorts this meta-analysis is likely underpowered to detect all outcome differences between HIPEC and non-HIPEC groups, and there is a risk of type II error occurring as a result. Further studies may consider evaluating the clinical significance of HIPEC versus non-HIPEC IH rates.

The proportion of patients developing an IH post CRS plus or minus HIPEC is in the range expected, considering midline laparotomies in general. This analysis suggested that HIPEC may contribute to a greater proportion of patients developing an IH, however, this finding was not statistically significant. Further studies may be clinically useful to further investigate HIPEC’s role in IH development.
